# Prevalence of intestinal trichomonads in captive non-human primates in China[Fn FN1]

**DOI:** 10.1051/parasite/2024018

**Published:** 2024-03-26

**Authors:** Ping-Ping Ma, Yang Zou, Wen-Jie Mu, Yue-Yue Zhang, Ya-Qi Li, Zhong-Li Liu, Long Zhang, Li-Xian Chen, Guo-Hua Liu, Shuai Wang

**Affiliations:** 1 State Key Laboratory for Animal Disease Control and Prevention, College of Veterinary Medicine, Lanzhou University, Lanzhou Veterinary Research Institute, Chinese Academy of Agricultural Sciences Lanzhou Gansu Province 730046 PR China; 2 Research Center for Parasites and Vectors, College of Veterinary Medicine, Hunan Agricultural University Changsha Hunan Province 410128 PR China

**Keywords:** Trichomonads, Prevalence, Non-human primates

## Abstract

Trichomonads are protozoan symbionts with the capacity to infect vertebrates including humans and non-human primates (NHPs), sometimes with pathogenic effects. However, their diversity and prevalence in NHPs in China are poorly understood. A total of 533 fecal samples were collected from captive NHPs in Yunnan Province, China, of which 461 samples from *Macaca fascicularis* and 72 from *Macaca mulatta*. Trichomonadidae species were identified using PCR amplification of the ITS-1/5.8S/ITS-2 sequences. The overall prevalence of trichomonads in NHPs was determined to be 11.4% (61/533), with gender, diarrhea, and region identified as potential risk factors for the infections. Sequence alignment and phylogenetic analysis identified three species of trichomonads, *i.e.*, *Trichomitopsis minor* (*n* = 45), *Pentatrichomonas hominis* (*n* = 11), and *Tetratrichomonas* sp. (*n* = 5). To the best of our knowledge, this is the first study to report *Trichomitopsis minor* infection in NHPs in China. Of note, *Pentatrichomonas hominis* is generally recognized as a parasitic organism affecting humans. Collectively, our results suggest that NHPs are potential sources of zoonotic trichomonad infections, highlighting the importance of surveillance and control measures to protect human and animal populations.

## Abstract (fr)

Les Trichomonadidae sont des symbiotes protozoaires capables d’infecter les vertébrés, notamment les humains et les primates non humains (PNH), parfois avec des effets pathogènes. Cependant, leur diversité et leur prévalence chez les PNH en Chine sont mal comprises. Au total, 533 échantillons fécaux ont été collectés sur des PNH captifs dans la province du Yunnan, en Chine, dont 461 échantillons de *Macaca fascicularis* et 72 de *Macaca mulatta*. Les espèces de Trichomonadidae ont été identifiées par amplification PCR des séquences ITS-1/5.8S/ITS-2. La prévalence globale des Trichomonadidae dans les PNH a été déterminée à 11,4 % (61 / 533) et le sexe, la diarrhée et la région ont été identifiés comme facteurs de risque potentiels d’infection. L’alignement des séquences et l’analyse phylogénétique ont identifié trois espèces de Trichomonadidae, à savoir *Trichomitopsis minor* (*n* = 45), *Pentatrichomonas hominis* (*n* = 11) et *Tetratrichomonas* sp. (*n* = 5). À notre connaissance, il s’agit de la première étude à signaler une infection par *Trichomitopsis minor* chez les PNH en Chine. Il convient de noter que *Pentatrichomonas hominis* est généralement reconnu comme un organisme parasitaire affectant les humains. Collectivement, nos résultats suggèrent que les PNH sont des sources potentielles d’infections zoonotiques à Trichomonadidae, soulignant l’importance des mesures de surveillance et de contrôle pour protéger les populations humaines et animales.

## Introduction

Trichomonads are zoonotic parasites with wide geographical distribution that inhabit the oral cavity, gastrointestinal tract, and urogenital tract of vertebrate and invertebrate hosts, transmitted through contaminated food or water via a fecal-oral route [21]. Infection may cause diarrhea, sterility, and vaginitis and many animals in close contact with humans are affected, such as cattle, pigs, dogs, cats, non-human primates (NHPs), and birds [1, 7, 14, 15, 23]. Analysis of the prevalence and diversity of trichomonad species in animals may facilitate the prevention and control of transmission to humans.

Trichomonads are detected through analysis of ITS1-5.8S rRNA-ITS2 and SSU rRNA gene sequences [5, 6]. Nine Trichomonad species have been detected in NHPs [23]. Some pathogenic species of trichomonads have also been recorded in humans and animals, such as *Trichomonas vaginalis* (*T. vaginalis*) in humans [10], *Trichomonas foetus* (*T. foetus*) in cattle [22], and *Trichomonas gallinae* (*T. gallinae*) in birds [1]. Other species have traditionally been regarded as commensal organisms in the host digestive tract, such as *Pentatrichomonas hominis* (*P. hominis*) and *Tetratrichomonas* sp., but may be pathogenic, causing diarrhea in some animals [11, 27]. *Pentatrichomonas hominis* infection has also more recently been associated with gastrointestinal cancer and may now be classified as a zoonotic pathogen rather than a symbiotic organism, pending further investigation of its pathogenicity [29].

NHPs share many genetic and physiological similarities with humans and are widely utilized in basic medical and life science research. Particularly in high-density, large-scale breeding centers, the close contact between NHPs and management staff may raise the risk of zoonotic transmission of trichomonads. However, the prevalence and species of trichomonads in NHPs in China has been little studied. The objective of this study was thus to determine the prevalence and species diversity of trichomonads in NHPs to better assess zoonotic potential.

## Material and methods

### Ethical standards

The research protocol was reviewed and approved by the Kunming Biomed International (KBI) Animal Experiment Management and Ethics Committee (No. KBI K001123083-01). Fecal samples were collected from *Macaca fascicularis* and *Macaca mulatta* with the permission of the breeders and managers. No animals were injured during the research.

### Sample collection

A total of 533 fecal samples were collected in Kunming city and Yuanjiang city in Yunnan Province, China between 2021 and 2022, including 72 from *Macaca mulatta* and 461 from *Macaca fascicularis*. The farm was cleaned the night before sampling, and each individual was caged separately so that the fresh feces of each individual was collected the next morning. To ensure the standardization of the fecal sample collection process and reduce potential pollution between samples, all fecal samples were collected by laboratory professionals. Only the middle layer of feces was collected during sample collection. Approximately 5–10 g of fresh fecal samples were collected immediately after defecation into a 5 mL collection tube with DNA preservation solution (Phygene Bio, Fuzhou, China), with cage number, gender, region, age, breed, and health condition recorded. All samples were put on ice and immediately transported to the laboratory for storage at −80 °C until processing.

### Extraction of genomic DNA

Samples were centrifuged at 8000 × *g* for 5 min to remove DNA storage solution and DNA extracted from 200 mg samples using an E.Z.N.A.^®^ stool DNA kit (OMEGA Bio-Tek Inc., Norcross, GA, USA), according to the manufacturer’s instructions. Extracted DNA was stored at −20 °C for PCR amplification.

### PCR amplification

The ITS-1/5.8S/ITS-2 genomic region was amplified using primers, NC5 forward primer: 5′-GTAGGTGAACCTGCGGAAGGATCATT-3′ and NC2 reverse primer: 5′-TTAGTTTCTTTTCCTCCGCT-3′ [13]. The amplification was carried out in 10 × PCR buffer, containing 2 μL genomic DNA, 0.2 mM dNTP mixture, 2 mM MgCl2, 0.625 U of Ex Taq (TaKaRa Bio Inc, Shanghai, China) and 1 μL primers with double-distilled water to a total volume of 25 μL. The following conditions were used for PCR amplification: initial denaturation at 94 °C for 4 min, 30 cycles of 94 °C for 30 s, 55 °C for 30 s, 72 °C for 30 s with a final extension at 72 °C for 10 min. Positive and negative controls were included and PCR products were subjected to 1.5% agarose gel electrophoresis and visualized under UV light. Positive PCR products were sequenced by TSING KE Biological Technology (Xi’an, China).

### Sequence alignment and phylogenetic analysis

Samples positive for trichomonads were identified by the basic local alignment search tool (BLAST) (http://www.ncbi.nlm.nih.gov/blast/) and aligned with published trichomonad reference sequences from GenBank, using ClustalX 2.0.11. A phylogenetic tree was constructed using the Maximum Likelihood method in MEGA 7.0 software with the Kimura 2 parametric model and reliability assessed through bootstrap analysis with 1,000 replicates.

### Statistical analysis

Statistical analysis was performed using IBM SPSS Statistics software (version 27). Differences in prevalence by age, gender, breed, health condition, and region were analyzed by chi-square (*χ*^2^) test. Odds ratios with 95% confidence intervals (CIs) were calculated. A *p*-value < 0.05 was considered statistically significant.

### Nucleotide sequence accession numbers

The representative nucleotide sequences were submitted to the GenBank database under accession numbers: OP353527–OP353530, OP778917–OP778930, and OP778932–OP778937.

## Results

### Prevalence of trichomonads in NHPs

Trichomonads were detected in 61 out of 533 fecal samples, resulting in a prevalence rate of 11.4%. The highest prevalence was observed in females with a rate of 16.0% (95% CI: 10.7–21.2) and that in males was 9.0% (95% CI: 6.0–12.0) (Table 1). The difference was statistically significant, indicating an association of gender with the probability of trichomonad infection. The infection rates of trichomonads in diarrhea and asymptomatic conditions were 25.6% (95% CI: 11.9–39.3) and 10.3% (95% CI: 7.6–13.0), respectively, and they showed a significant difference (*p* < 0.01) (Table 1). In addition, a significant difference was found among different regions (*p* < 0.001). The highest prevalence was observed in Yuanjiang city with a rate of 14.3% (95% CI: 10.9–17.6), and that in Kunming city was 0.9% (95% CI: 0–2.6) (Table 1). Of note, trichomonads were not detected in suckling monkeys (< 2 years) (Table 1).

**Table 1:** Factors associated with the prevalence of trichomonads in two non-human primate species.

Factor	Group	No. positive/Sample size	Prevalence (%) (95% CI)	OR (95% CI)	*p*-value	Species
Age	<2 years	0/24	0	–	–	
	2–3 years	18/176	10.2 (5.8–14.7)	–		*T. minor* (*n* = 15), *P. hominis* (*n* = 2), *Tetratrichomonas* sp. (*n* = 1)
	> 3 years	43/333	12.9 (9.3–16.5)	–		*T. minor* (*n* = 30), *P. hominis* (*n* = 9), *Tetratrichomonas* sp. (*n* = 4)
Gender	Male	31/345	9.0 (6.0–12.0)	1	<0.05	*T. minor* (*n* = 19), *P. hominis* (*n* = 7), *Tetratrichomonas* sp. (*n* = 5)
	Female	30/188	16.0 (10.7–21.2)	1.9 (1.1–3.3)		*T. minor* (*n* = 26), *P. hominis* (*n* = 4)
Breed	*Macaca fascicularis*	49/461	10.6 (7.8–13.4)	1	0.13	*T. minor* (*n* = 36), *P. hominis* (*n* = 8), *Tetratrichomonas* sp. (*n* = 5)
	*Macaca mulatta*	12/72	16.7 (8.1–25.3)	1.7 (0.8–3.3)		*T. minor* (*n* = 9), *P. hominis* (*n* = 3)
Clinical symptoms	Asymptomatic	51/494	10.3 (7.6–13.0)	1	<0.01	*T. minor* (*n* = 38), *P. hominis* (*n* = 8), *Tetratrichomonas* sp. (*n* = 5)
	Diarrhea	10/39	25.6 (11.9–39.3)	3 (1.4–6.5)		*T. minor* (*n* = 7), *P. hominis* (*n* = 3)
Region	Kunming city	1/113	0.9 (0–2.6)	1	<0.001	*Tetratrichomonas* sp. (*n* = 1)
	Yuanjiang city	60/420	14.3 (10.9–17.6)	18.7 (2.6–136.2)		*T. minor* (*n* = 45), *P. hominis* (*n* = 11), *Tetratrichomonas* sp. (*n* = 4)
Total	–	61/533	11.4 (8.7–14.1)	–	–	*T. minor* (*n* = 45), *P. hominis* (*n* = 11), *Tetratrichomonas* sp. (*n* = 5)

95% CI: 95% confidence interval; OR: odds ratio.

### Species identification

Analysis of ITS-1/5.8S/ITS-2 sequences indicated the presence of three trichomonad species in 61 positive samples, including *Trichomitopsis minor* (*T. minor*) (*n* = 45), *P. hominis* (*n* = 11), and *Tetratrichomonas* sp. (*n* = 5). *Trichomitopsis minor* was predominant, accounting for 73.8% of samples (45 out of 61). *Pentatrichomonas hominis*, which is recognized as a human pathogen, was present in 11 samples. *Tetratrichomonas* sp. was present in five samples and requires further characterization to assign a taxonomic classification. No mixed infections of multiple trichomonad species were detected.

### Phylogenetic analysis

**Figure 1:**
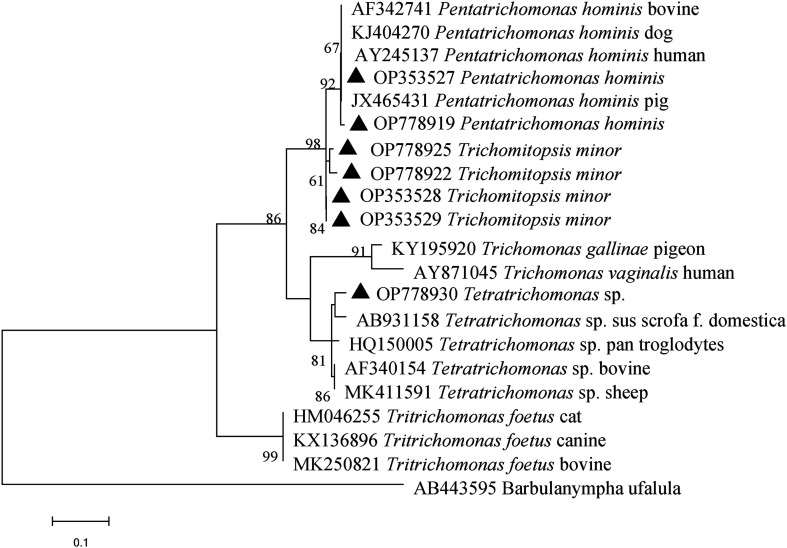
Phylogenetic analysis of trichomonad gene subtypes based on ITS region sequence using the Maximum Likelihood method. The bootstrap value was determined with 1,000 replicates, and values lower than 60% are not displayed. Trichomonad isolates identified in this study are indicated by solid black triangles.

Phylogenetic analysis revealed clustering of species isolates, OP353527 and OP778919, which belong to *P. hominis* (Fig. 1). These isolates clustered with those derived from pigs, humans, dogs, and cattle. The isolates of *T. minor*, OP353528, OP353529, OP778922, and OP778925, clustered together in a single branch which showed a close genetic relationship with the *P. hominis* isolates. *Tetratrichomonas* sp. isolates, particularly OP778930, were distinct and showed a distant genetic relationship with *P. hominis*.

## Discussion

Trichomonads are common intestinal protozoan symbionts with a global distribution that can infect a range of hosts; wild and captive animals serve as potential reservoirs for human infection [8, 19, 20]. Limited work has been undertaken on trichomonad infection in NHPs and prevalence, genetic diversity, and pathogenic potential are poorly understood. The present study found overall trichomonad prevalence in NHPs to be 11.4% (61/533), lower than findings previously reported in NHPs in North China (46.7%, 28/60) [18], gorillas in the Central African Republic (27.6%, 45/163) [24], wild chimpanzees in Uganda (68.6%, 48/70), and NHPs in Southeastern Brazil (31.3%, 5/16) [9, 25]. Differences in prevalence may be due to the influence of multiple risk factors governing infection and sample sizes have generally been small, at fewer than 100 subjects [9, 18, 25]. Prevalence rates are associated with the specific population under study and may not be generalized to all NHP populations. Further research is necessary to determine the impact of NHP species and age on trichomonads prevalence and diversity.

Gender is identified as a risk factor for trichomonads infection. Previous research has indicated a higher prevalence of *P. hominis* infection in men compared to women [29]. Similarly, findings in foxes showed a higher incidence of *P. hominis* infection in males compared to females [26]. Nevertheless, in the current study, trichomonad infection in females surpassed that in males. These variations may stem from the combined effects of multiple factors, warranting further research to elucidate the specific reasons for this observed difference in the present study. Moreover, trichomonad infection has previously been linked to diarrhea [3, 16, 17]. Our results showed that diarrhea was closely related to trichomonad infection, and a higher prevalence was associated with the presence of diarrhea (25.6%) than with its absence (10.3%) (Table 1). In this study, we did not detect trichomonads in suckling monkeys (<2 years old) (Table 1). It is speculated that suckling monkeys may obtain maternal antibodies through milk to resist trichomonad infection [4].

The prevalence rate of trichomonads in different geographical areas was significantly different in the present study. The prevalence of trichomonads in Yuanjiang city (14.3%) was significantly higher than that in Kunming city (0.9%) (*p* < 0.001) (Table 1), similar to results reported in another study [16]. The differences in trichomonad prevalence in different geographical areas may be related to the management model and sanitary conditions of farms. The sanitary conditions are relatively poor and there is a lack of good immunization procedures in Yuanjiang city. In addition, the number of samples collected in different geographical areas is different, which may also be one of the reasons for the differences in trichomonad prevalence.

Three trichomonad species were identified in the current work, *T. minor*, *P. hominis,* and *Tetratrichomonas* sp. (Table 1), and *P. hominis* and *Tetratrichomonas* sp. have previously been documented in NHPs [2, 18, 24]. Importantly, *P. hominis* has a wide range of hosts and has frequently been detected in many mammals including humans, highlighting its zoonotic potential [12, 28]. However, due to the lack of data regarding the study of *P. hominis* from the investigated areas, the transmission routes of NHPs with *P. hominis* infection could not be elucidated in this study. The *Tetratrichomonas* sp. detected had distant genetic relationships with *P. hominis*, highlighting trichomonad genetic variability. To the best of our knowledge, this is the first study to report *T. minor* infection in NHPs in China. Further studies are required to elucidate transmission characteristics.

## Conclusion

The prevalence of trichomonad infection in NHPs in Yunnan Province, China was 11.4%. Three trichomonad species were identified, including a subtype with zoonotic potential. *Trichomitopsis minor* was detected in NHPs for the first time. Gender, diarrhea, and region have been identified as risk factors for trichomonad infection. Data are presented on the prevalence, genetic diversity, and zoonotic potential of trichomonads in NHPs to facilitate infection prevention measures.
